# Supplementary material for: The Evolution and Characterization of the RNA Interference Pathways in Lophotrochozoa
Source: Genome Biol Evol. 2024 May 7;16(5):evae098. doi: 10.1093/gbe/evae098 (PMC11114477; doi:10.1093/gbe/evae098)
Supplement: evae098_Supplementary_Data [file evae098_supplementary_data.zip › Supplementary_materials.zip!/selected_miRNAs/NoGe_selected_miRNAs/mirdeep_pdf_files/NMRB01000819.1_Notospermus_geniculatus_strain_Ushimado_scaffold819,_whole_genome_shotgun_sequence_21653.pdf]

Provisional ID	:	NMRB01000819.1_Notospermus_geniculatus_strain_Ushimado_scaffold819,_whole_genome_shotgun_sequence_21653
Score total	:	80990
Score for star read(s)	:	3.9
Score for read counts	:	80982.8
Score for mfe	:	0.3
Score for randfold	:	0
Score for cons. seed	:	3
Total read count	:	158856
Mature read count	:	158457
Loop read count	:	0
Star read count	:	399

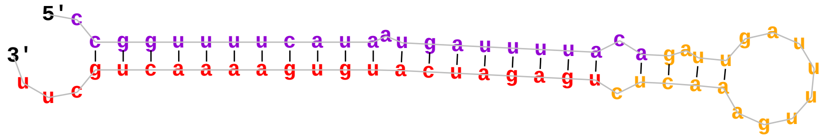

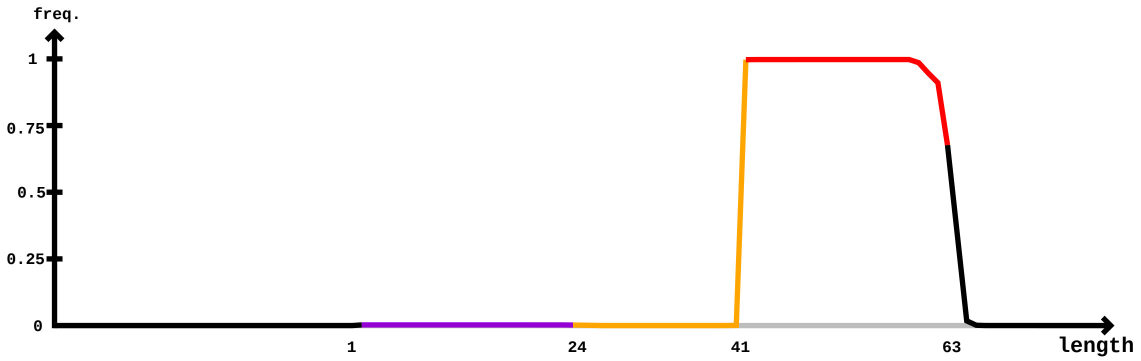

Star Mature

```
5'- ggacauggagcucuaccauaaaagacgaaaccgguuuucauaaugauuuuacagauugauuuugaaacucugagaucaugugaaaacugcuuuugucgacagugauca   -3'   obs
    ggacauggagcucuaccauaaaagacgaaaccgguuuucauaaugauuuuacagauugauuuugaaacucugagaucaugugaaaacugcuuuugucgacagugauca         exp
    ....((((.......))))....(((((((.(((((((((((.((((((((.((.((........)))).))))))))))))))))))).)))))))((......)).  reads   mm   sample
    ..............................ccgguuuucauaaugauu............................................................  1       0    Mm3
    ..............................ccgguuuucauaaugauuuuac........................................................  9       0    Mm3
    ..............................ccgguuuucauaaugauuuuaca.......................................................  6       0    Mm3
    ..............................ccgguuuucauaaugauuuuacag......................................................  2       0    Mm3
    ..............................ccgguuuucauaaugauuuuacaga.....................................................  3       0    Mm3
    ..............................ccgguuuucauaaugauuuuacagauug..................................................  1       0    Mm3
    .....................................................................cugagaucaugugaaaacugcu.................  6       0    Mm3
    .....................................................................cugagaucaugugaaaacugcuu................  5       0    Mm3
    .....................................................................cugagaucaugugaaaacugcuuu...............  2       0    Mm3
    ......................................................................ugagaucaugugaaaacG....................  1       1    Mm3
    ......................................................................ugagaucaugugaaaacu....................  96      0    Mm3
    ......................................................................ugagaucaugugaaaacC....................  1       1    Mm3
    ......................................................................ugagaucaugugaaaacCg...................  1       1    Mm3
    ......................................................................ugagaucaugugaaaacuA...................  14      1    Mm3
    ......................................................................ugagaucaugugaaaacug...................  122     0    Mm3
    ......................................................................ugagaucaugugaaaaAug...................  1       1    Mm3
    ......................................................................ugagaucaugugaaaacuU...................  5       1    Mm3
    ......................................................................ugagaucaugugaaaacAgc..................  1       1    Mm3
    ......................................................................ugagauAaugugaaaacugc..................  1       1    Mm3
    ......................................................................ugagaucaugugaaaaAugc..................  6       1    Mm3
    ......................................................................ugagaucaugugaaaacCgc..................  3       1    Mm3
    ......................................................................Ngagaucaugugaaaacugc..................  1       1    Mm3
    ......................................................................ugagaucaugugaaaacGgc..................  2       1    Mm3
    ......................................................................ugagaucaugugaaaacugc..................  579     0    Mm3
    ......................................................................ugagaucaugCgaaaacugc..................  1       1    Mm3
    ......................................................................ugagaucaugugaaaacugU..................  8       1    Mm3
    ......................................................................ugagaucaugugGaaacugc..................  1       1    Mm3
    ......................................................................ugagaucauAugaaaacugcu.................  1       1    Mm3
    ......................................................................ugagaucaugugaaaacAgcu.................  29      1    Mm3
    ......................................................................ugagaucaugGgaaaacugcu.................  3       1    Mm3
    ......................................................................ugagaucaugugaaaacugcC.................  5       1    Mm3
    ......................................................................ugagaNcaugugaaaacugcu.................  1       1    Mm3
    ......................................................................ugagaucaugugaaaGcugcu.................  1       1    Mm3
```

Star **Mature**

```
ggacauggagcucuaccauaaaagacgaaaccgguuuucauaaugauuuuacagauugauuuugaaacucugagaucaugugaaaacugcuuuugucgacagugauca
......................................................................ugagaucaugugaaaacugcA.................  23    1    Mm3
......................................................................ugagaucaugugaaaacGgcu.................  24    1    Mm3
......................................................................ugagaucaugugaaUacugcu.................  3     1    Mm3
......................................................................ugagaucaugugaUaacugcu.................  1     1    Mm3
......................................................................ugagaucaugugaaaacugcG.................  11    1    Mm3
......................................................................ugagaucGugugaaaacugcu.................  3     1    Mm3
......................................................................Agagaucaugugaaaacugcu.................  1     1    Mm3
......................................................................ugagaucaugAgaaaacugcu.................  12    1    Mm3
......................................................................ugagaucaugugaaaaGugcu.................  5     1    Mm3
......................................................................ugGgaucaugugaaaacugcu.................  1     1    Mm3
......................................................................ugagaucaugugaaaaAugcu.................  22    1    Mm3
......................................................................uAagaucaugugaaaacugcu.................  2     1    Mm3
......................................................................ugagCucaugugaaaacugcu.................  2     1    Mm3
......................................................................ugagaucaugugaaGacugcu.................  2     1    Mm3
......................................................................ugagaucaugugaaaacugcu.................  5317  0    Mm3
......................................................................ugagaucaugugaaaacCgcu.................  27    1    Mm3
......................................................................Cgagaucaugugaaaacugcu.................  1     1    Mm3
......................................................................ugaAaucaugugaaaacugcu.................  1     1    Mm3
......................................................................ugagaucaCgugaaaacugcu.................  1     1    Mm3
......................................................................ugagaucaugugaGaacugcu.................  1     1    Mm3
......................................................................ugagaucaugugaCaacugcu.................  1     1    Mm3
......................................................................ugagaucaugugaaaacugUu.................  3     1    Mm3
......................................................................ugagaucaugCgaaaacugcu.................  10    1    Mm3
......................................................................ugagaucaugugaaaUcugcu.................  1     1    Mm3
......................................................................ugagaucaugugaaaaUugcu.................  13    1    Mm3
......................................................................Ngagaucaugugaaaacugcu.................  2     1    Mm3
......................................................................ugagauUaugugaaaacugcu.................  2     1    Mm3
......................................................................ugagaucauguUaaaacugcu.................  1     1    Mm3
......................................................................ugagaucaugugaaaacugAuu................  3     1    Mm3
......................................................................uCagaucaugugaaaacugcuu................  1     1    Mm3
......................................................................ugagaNcaugugaaaacugcuu................  2     1    Mm3
......................................................................ugagaAcaugugaaaacugcuu................  2     1    Mm3
......................................................................ugagaucauguCaaaacugcuu................  1     1    Mm3
......................................................................ugagaucaugGgaaaacugcuu................  10    1    Mm3
......................................................................Cgagaucaugugaaaacugcuu................  1     1    Mm3
......................................................................ugagaucauguUaaaacugcuu................  2     1    Mm3
......................................................................ugagaucaAgugaaaacugcuu................  1     1    Mm3
......................................................................ugagaucaugugaaaaGugcuu................  14    1    Mm3
......................................................................Ngagaucaugugaaaacugcuu................  8     1    Mm3
......................................................................ugagaucaugugaUaacugcuu................  1     1    Mm3
......................................................................ugagaucaugugaaaacugcCu................  10    1    Mm3
......................................................................ugagaucaugugaaaCcugcuu................  3     1    Mm3
......................................................................ugNgaucaugugaaaacugcuu................  1     1    Mm3
......................................................................ugagaucaugCgaaaacugcuu................  10    1    Mm3
......................................................................ugagaucaugugaaaacuUcuu................  2     1    Mm3
......................................................................ugagUucaugugaaaacugcuu................  2     1    Mm3
......................................................................ugagaucauCugaaaacugcuu................  2     1    Mm3
......................................................................ugagaucUugugaaaacugcuu................  3     1    Mm3
......................................................................ugagaucaugugUaaacugcuu................  2     1    Mm3
......................................................................ugagaucaugugaaaacugcAu................  2     1    Mm3
......................................................................ugagaucaugugaaaaAugcuu................  58    1    Mm3
......................................................................ugagaucaugugaaaacugcuA................  27    1    Mm3
......................................................................ugagaucaugugaaaacugcuG................  5     1    Mm3
......................................................................ugagaucaugugaaaUcugcuu................  1     1    Mm3
......................................................................ugaAaucaugugaaaacugcuu................  1     1    Mm3
......................................................................ugagaucaugugaaaacugcuC................  1     1    Mm3
......................................................................ugaUaucaugugaaaacugcuu................  1     1    Mm3
......................................................................ugagaucaugugCaaacugcuu................  1     1    Mm3
......................................................................uAagaucaugugaaaacugcuu................  5     1    Mm3
......................................................................ugagaucaugugaaaacugGuu................  1     1    Mm3
......................................................................ugagaucaugugaaaaUugcuu................  24    1    Mm3
......................................................................ugagaucaugugaaaacuAcuu................  1     1    Mm3
......................................................................ugagaucauguAaaaacugcuu................  2     1    Mm3
......................................................................ugagauAaugugaaaacugcuu................  1     1    Mm3
......................................................................Agagaucaugugaaaacugcuu................  2     1    Mm3
......................................................................ugagaucaugugaaaacGgcuu................  60    1    Mm3
......................................................................ugagauNaugugaaaacugcuu................  2     1    Mm3
......................................................................ugagaucaugugaaUacugcuu................  1     1    Mm3
......................................................................ugagaucaugugaaaacugUuu................  10    1    Mm3
......................................................................ugagaucaugugaaaacAgcuu................  82    1    Mm3
```

Star **Mature**

```
ggacauggagcucuaccauaaaagacgaaaccgguuuucauaaugauuuuacagauugauuuugaaacucugagaucaugugaaaacugcuuuugucgacagugauca
......................................................................ugagCucaugugaaaacugcuu................ 6      1  Mm3
......................................................................ugagaucaugugaCaacugcuu................ 1      1  Mm3
......................................................................ugagaucaugugaaaacCgcuu................ 81     1  Mm3
......................................................................ugGgaucaugugaaaacugcuu................ 10     1  Mm3
......................................................................ugagGucaugugaaaacugcuu................ 3      1  Mm3
......................................................................ugagaucaugugaaaacugcuu................ 13768  0  Mm3
......................................................................ugagaucaugugaaaGcugcuu................ 1      1  Mm3
......................................................................ugagaucaugugaGaacugcuu................ 4      1  Mm3
......................................................................Ggagaucaugugaaaacugcuu................ 1      1  Mm3
......................................................................ugagaucaugugGaaacugcuu................ 1      1  Mm3
......................................................................ugagaucaugAgaaaacugcuu................ 33     1  Mm3
......................................................................ugagauUaugugaaaacugcuu................ 5      1  Mm3
......................................................................ugagaucaugugaaGacugcuu................ 1      1  Mm3
......................................................................uUagaucaugugaaaacugcuu................ 1      1  Mm3
......................................................................ugagaucauUugaaaacugcuu................ 3      1  Mm3
......................................................................ugagaucGugugaaaacugcuu................ 4      1  Mm3
......................................................................ugCgaucaugugaaaacugcuu................ 1      1  Mm3
......................................................................uAagaucaugugaaaacugcuuu............... 1      1  Mm3
......................................................................ugagaucaugugaaaaUugcuuu............... 4      1  Mm3
......................................................................ugagaucaugugaaaacugcuGu............... 1      1  Mm3
......................................................................ugagaucaugugaaaacugcuuG............... 1      1  Mm3
......................................................................ugagaucUugugaaaacugcuuu............... 1      1  Mm3
......................................................................ugagCucaugugaaaacugcuuu............... 1      1  Mm3
......................................................................uNagaucaugugaaaacugcuuu............... 1      1  Mm3
......................................................................ugagaucaugugaaaacugcCuu............... 1      1  Mm3
......................................................................ugagaucaugugaaaacGgcuuu............... 10     1  Mm3
......................................................................ugagaucaugugaaaaAugcuuu............... 6      1  Mm3
......................................................................ugagaucaugugaaaacAgcuuu............... 8      1  Mm3
......................................................................ugagaucaugGgaaaacugcuuu............... 2      1  Mm3
......................................................................ugagaucaugugaGaacugcuuu............... 1      1  Mm3
......................................................................ugagaucaugCgaaaacugcuuu............... 1      1  Mm3
......................................................................ugagaucaugugaaUacugcuuu............... 1      1  Mm3
......................................................................ugagaucaugugaaaacugcGuu............... 1      1  Mm3
......................................................................ugagaucaugugaaaacugcuuA............... 122    1  Mm3
......................................................................ugagGucaugugaaaacugcuuu............... 1      1  Mm3
......................................................................ugagaucaugugaaaacugcuuC............... 2      1  Mm3
......................................................................ugagaucaugugaaaacugcuuu............... 1662   0  Mm3
......................................................................ugagaucaugugaaaacCgcuuu............... 8      1  Mm3
......................................................................ugagaucaugugaaaacugcuAu............... 2      1  Mm3
......................................................................ugagaucaugAgaaaacugcuuu............... 7      1  Mm3
......................................................................ugagaucaCgugaaaacugcuuuu.............. 1      1  Mm3
......................................................................ugagaucaugugaaaacCgcuuuu.............. 1      1  Mm3
......................................................................ugUgaucaugugaaaacugcuuuu.............. 1      1  Mm3
......................................................................ugagaucaugugaaaacAgcuuuu.............. 3      1  Mm3
......................................................................ugagaucaugugaaaacugcuuuA.............. 9      1  Mm3
......................................................................ugGgaucaugugaaaacugcuuuu.............. 1      1  Mm3
......................................................................ugagaucaugugaaaacGgcuuuu.............. 2      1  Mm3
......................................................................ugagaucaugugaaaacugcuuAu.............. 7      1  Mm3
......................................................................ugagaucaugugaaaacugcuuuu.............. 213    0  Mm3
......................................................................ugagaucaugugaaaacugcuuuuU............. 26     1  Mm3
......................................................................ugagaucaugugaaaacugcuuuuCu............ 1      1  Mm3
......................................................................ugagaucaugugaaaacugcuuuuUu............ 5      1  Mm3
.......................................................................gagaucaugugaaaacugc.................. 1      0  Mm3
.......................................................................gagaucaugugaaaacugcu................. 2      0  Mm3
.......................................................................gagaucaugugaaaacugcuu................ 6      0  Mm3
.......................................................................gagaucaugugaaaacugcuuu............... 2      0  Mm3
........................................................................agaucaugugaaaacugcuu................ 1      0  Mm3

..............................ccgguuuucauaaugauA............................................................ 1      1  Mm2
..............................ccgguuuucauaaugauuuu.......................................................... 2      0  Mm2
..............................ccgguuuucauaaugauuuuac........................................................ 5      0  Mm2
..............................ccgguuuucauaaugauuuuaca....................................................... 10     0  Mm2
..............................ccgguuuucauaaugauuuuacag...................................................... 2      0  Mm2
..............................ccgguuuucauaaugauuuuacaga..................................................... 9      0  Mm2
..............................Ncgguuuucauaaugauuuuacaga..................................................... 1      1  Mm2
...................................................................cucugagaucaugugaaaacugcu................. 1      0  Mm2
....................................................................ucugagaucaugugaaaacugc.................. 1      0  Mm2
....................................................................ucugagaucaugugaaaacugcu................. 1      0  Mm2
.....................................................................cugagaucaugugaaaacugcu................. 11     0  Mm2
.....................................................................cugagaucaugugaaUacugcuu................ 1      1  Mm2
```

```
                                              Star                                       Mature
ggacauggagcucuaccauaaaagacgaaaccgguuuucauaaugauuuuacagauugauuuugaaacucugagaucaugugaaaacugcuuuugucgacagugauca
.....................................................................cugagaucaugugaaaacugcuuu...............   1      0    Mm2
.....................................................................cugagaucaugugaaaacugcuuuu..............   2      0    Mm2
......................................................................ugagaucaugugaaaaAu....................   1      1    Mm2
......................................................................ugagaucaugugaaaacu....................   106    0    Mm2
......................................................................ugagaucaugugaaaacG....................   1      1    Mm2
......................................................................ugagaucaugugaaaacuA...................   15     1    Mm2
......................................................................ugagaucaugugaaaacuU...................   3      1    Mm2
......................................................................ugagaucaugugaaaacCg...................   2      1    Mm2
......................................................................ugagaucaugugaaaacug...................   213    0    Mm2
......................................................................ugagaucaugugaaaaUug...................   1      1    Mm2
......................................................................ugagaucaugugaaaacAg...................   1      1    Mm2
......................................................................ugagaucUugugaaaacug...................   2      1    Mm2
......................................................................ugagaucaugugaaaacGg...................   1      1    Mm2
......................................................................ugagaucaugugaaaaAug...................   1      1    Mm2
......................................................................ugagaucaugugaaaaUugc..................   2      1    Mm2
......................................................................ugagGucaugugaaaacugc..................   1      1    Mm2
......................................................................ugUgaucaugugaaaacugc..................   1      1    Mm2
......................................................................ugagaucaugugaaaacugU..................   3      1    Mm2
......................................................................ugagaucaugAgaaaacugc..................   1      1    Mm2
......................................................................ugagaucaugugaaaacCgc..................   4      1    Mm2
......................................................................ugagaucaugugaaaacugG..................   1      1    Mm2
......................................................................ugagaucaugugaaaaAugc..................   3      1    Mm2
......................................................................ugagaucaugCgaaaacugc..................   1      1    Mm2
......................................................................ugagaucaugugaaaacugc..................   620    0    Mm2
......................................................................ugagaucaugugaaaacAgc..................   5      1    Mm2
......................................................................ugagaucaugugaaaacGgc..................   4      1    Mm2
......................................................................ugagaCcaugugaaaacugcu.................   1      1    Mm2
......................................................................ugGgaucaugugaaaacugcu.................   1      1    Mm2
......................................................................ugagaucaugugaaaacugcG.................   12     1    Mm2
......................................................................Cgagaucaugugaaaacugcu.................   2      1    Mm2
......................................................................Agagaucaugugaaaacugcu.................   1      1    Mm2
......................................................................ugagaucaugGgaaaacugcu.................   4      1    Mm2
......................................................................ugagaucaugugaaaaAugcu.................   12     1    Mm2
......................................................................ugagaucaugugaaaaUugcu.................   7      1    Mm2
......................................................................ugagaucaugugaaaacCgcu.................   22     1    Mm2
......................................................................ugagaucauCugaaaacugcu.................   1      1    Mm2
......................................................................ugagaucaugugaaaacugcA.................   18     1    Mm2
......................................................................ugagaucaugugUaaacugcu.................   1      1    Mm2
......................................................................ugagaucaugugaaGacugcu.................   1      1    Mm2
......................................................................uAagaucaugugaaaacugcu.................   2      1    Mm2
......................................................................ugagaucauguUaaaacugcu.................   1      1    Mm2
......................................................................ugagaucaugugaaaacGgcu.................   29     1    Mm2
......................................................................ugagaucaugugaUaacugcu.................   1      1    Mm2
......................................................................ugagaucaugugaaaacugUu.................   2      1    Mm2
......................................................................ugagaucaugugaaaacugAu.................   1      1    Mm2
......................................................................ugagaucaugugaaaacAgcu.................   25     1    Mm2
......................................................................ugagaucaugCgaaaacugcu.................   5      1    Mm2
......................................................................ugagaucaugugaaaacugcC.................   3      1    Mm2
......................................................................ugagaucaugAgaaaacugcu.................   10     1    Mm2
......................................................................ugagaucaCgugaaaacugcu.................   1      1    Mm2
......................................................................ugCgaucaugugaaaacugcu.................   1      1    Mm2
......................................................................ugagUucaugugaaaacugcu.................   1      1    Mm2
......................................................................Ngagaucaugugaaaacugcu.................   3      1    Mm2
......................................................................ugagaucUugugaaaacugcu.................   3      1    Mm2
......................................................................ugagaucaugugaaaUcugcu.................   1      1    Mm2
......................................................................ugagaucaugugaaaacugcu.................   4932   0    Mm2
......................................................................ugagaucaugugaGaacugcu.................   3      1    Mm2
......................................................................ugagaucaugugaaaGcugcu.................   1      1    Mm2
......................................................................ugagGucaugugaaaacugcu.................   1      1    Mm2
......................................................................ugagaucauAugaaaacugcu.................   1      1    Mm2
......................................................................ugagaAcaugugaaaacugcu.................   1      1    Mm2
......................................................................ugagaucaugugaaaacuUcu.................   1      1    Mm2
......................................................................ugagaucaugugaaaaGugcu.................   6      1    Mm2
......................................................................ugagUucaugugaaaacugcuu................   1      1    Mm2
......................................................................ugagaucaugugaaaaUugcuu................   40     1    Mm2
......................................................................ugagauAaugugaaaacugcuu................   1      1    Mm2
......................................................................ugagaucaugugaaUacugcuu................   1      1    Mm2
......................................................................ugagaAcaugugaaaacugcuu................   3      1    Mm2
......................................................................ugagaucaugugaaaaGugcuu................   19     1    Mm2
......................................................................ugagaucaugugaaCacugcuu................   1      1    Mm2
```

Star **Mature**

```
ggacauggagcucuaccauaaaagacgaaaccgguuuucauaaugauuuuacagauugauuuugaaacucugagaucaugugaaaacugcuuuugucgacagugauca
......................................................................Ngagaucaugugaaaacugcuu................ 7 1 Mm2
......................................................................ugagaucaugCgaaaacugcuu................ 17 1 Mm2
......................................................................ugagaucaugugaaaacugcuu................ 18640 0 Mm2
......................................................................ugagGucaugugaaaacugcuu................ 2 1 Mm2
......................................................................ugagaucaugugaaGacugcuu................ 1 1 Mm2
......................................................................ugagaucaugugaaaacugcCu................ 8 1 Mm2
......................................................................ugagaucaAgugaaaacugcuu................ 1 1 Mm2
......................................................................ugagaucauguAaaaacugcuu................ 1 1 Mm2
......................................................................ugagaucaugugaaaacugcGu................ 2 1 Mm2
......................................................................ugagaNcaugugaaaacugcuu................ 2 1 Mm2
......................................................................ugagauUaugugaaaacugcuu................ 6 1 Mm2
......................................................................ugagaucaugugaaaacugcuC................ 6 1 Mm2
......................................................................ugagaucauAugaaaacugcuu................ 3 1 Mm2
......................................................................ugUgaucaugugaaaacugcuu................ 3 1 Mm2
......................................................................ugagaucaugugaaaacuCcuu................ 1 1 Mm2
......................................................................uUagaucaugugaaaacugcuu................ 3 1 Mm2
......................................................................ugagaucaugugaaaacugAuu................ 2 1 Mm2
......................................................................ugagaucaugugaaaacuAcuu................ 1 1 Mm2
......................................................................uCagaucaugugaaaacugcuu................ 1 1 Mm2
......................................................................ugagaucaugGgaaaacugcuu................ 23 1 Mm2
......................................................................uAagaucaugugaaaacugcuu................ 6 1 Mm2
......................................................................ugagaucauguUaaaacugcuu................ 2 1 Mm2
......................................................................ugagaucaugugaaaacAgcuu................ 92 1 Mm2
......................................................................ugagCucaugugaaaacugcuu................ 1 1 Mm2
......................................................................ugaAaucaugugaaaacugcuu................ 2 1 Mm2
......................................................................Agagaucaugugaaaacugcuu................ 7 1 Mm2
......................................................................ugagaucauguCaaaacugcuu................ 2 1 Mm2
......................................................................ugagaucaugugaaaacCgcuu................ 112 1 Mm2
......................................................................ugagaucaugugUaaacugcuu................ 1 1 Mm2
......................................................................ugagaucaugugaCaacugcuu................ 1 1 Mm2
......................................................................ugCgaucaugugaaaacugcuu................ 2 1 Mm2
......................................................................ugagaucaugugaaaacugcuA................ 22 1 Mm2
......................................................................ugagaucaugugGaaacugcuu................ 3 1 Mm2
......................................................................ugagaucaugugaaaGcugcuu................ 1 1 Mm2
......................................................................ugagaucUugugaaaacugcuu................ 2 1 Mm2
......................................................................ugagaCcaugugaaaacugcuu................ 3 1 Mm2
......................................................................ugagaucaugugaaaacGgcuu................ 93 1 Mm2
......................................................................ugagaucGugugaaaacugcuu................ 3 1 Mm2
......................................................................ugagaucaugugaaaUcugcuu................ 4 1 Mm2
......................................................................ugagaucaugugaaaacugUuu................ 9 1 Mm2
......................................................................ugGgaucaugugaaaacugcuu................ 4 1 Mm2
......................................................................ugagaucaCgugaaaacugcuu................ 3 1 Mm2
......................................................................ugagaucaugugaaaacuUcuu................ 8 1 Mm2
......................................................................Cgagaucaugugaaaacugcuu................ 4 1 Mm2
......................................................................ugagaucaugugaaaaAugcuu................ 77 1 Mm2
......................................................................ugagaucaugugaUaacugcuu................ 2 1 Mm2
......................................................................ugagaucaugugaaaacugcuG................ 11 1 Mm2
......................................................................Ggagaucaugugaaaacugcuu................ 1 1 Mm2
......................................................................ugagaucaugugaGaacugcuu................ 4 1 Mm2
......................................................................ugagaucaugAgaaaacugcuu................ 33 1 Mm2
......................................................................ugagaucaugugaaaacugcuGu............... 1 1 Mm2
......................................................................ugagaucaugugaaaacugcuuC............... 9 1 Mm2
......................................................................ugagaucaugugaaaacugcuuu............... 2484 0 Mm2
......................................................................ugagaucaugugaaaaGugcuuu............... 6 1 Mm2
......................................................................ugagaucaugugaaGacugcuuu............... 1 1 Mm2
......................................................................uCagaucaugugaaaacugcuuu............... 2 1 Mm2
......................................................................ugagaucaugugaaaacAgcuuu............... 10 1 Mm2
......................................................................ugagaucaCgugaaaacugcuuu............... 1 1 Mm2
......................................................................ugaAaucaugugaaaacugcuuu............... 1 1 Mm2
......................................................................ugagaucauAugaaaacugcuuu............... 1 1 Mm2
......................................................................ugagaucaugugaaaaUugcuuu............... 6 1 Mm2
......................................................................ugagaucaugugaaaacugcuuG............... 1 1 Mm2
......................................................................ugagaucaugugaaaacugcuuA............... 159 1 Mm2
......................................................................Ngagaucaugugaaaacugcuuu............... 3 1 Mm2
......................................................................ugagaucaugAgaaaacugcuuu............... 4 1 Mm2
......................................................................ugagaucaugugaaUacugcuuu............... 2 1 Mm2
......................................................................ugCgaucaugugaaaacugcuuu............... 1 1 Mm2
......................................................................ugagaucaGgugaaaacugcuuu............... 1 1 Mm2
......................................................................ugagaucaugugaaaacCgcuuu............... 9 1 Mm2
......................................................................ugagaucaugCgaaaacugcuuu............... 3 1 Mm2
```

Star / Mature

```
                              Star                                  Mature
ggacauggagcucuaccauaaaagacgaaaccgguuuucauaaugauuuuacagauugauuuugaaacucugagaucaugugaaaacugcuuuugucgacagugauca
......................................................................Ggagaucaugugaaaacugcuu................    1     1    Mm2
......................................................................ugagaucaugugaaaacugUuuu...............    2     1    Mm2
......................................................................ugagaucaugugaaaacugcCuu...............    4     1    Mm2
......................................................................Cgagaucaugugaaaacugcuu................    1     1    Mm2
......................................................................ugGgaucaugugaaaacugcuu................    1     1    Mm2
......................................................................ugagauUaugugaaaacugcuu................    1     1    Mm2
......................................................................ugagaucaugugaaaacGgcuu................    11    1    Mm2
......................................................................ugagaucaugugaaaaAugcuu................    18    1    Mm2
......................................................................ugagaucaugGgaaaacugcuu................    2     1    Mm2
......................................................................ugagauGaugugaaaacugcuu................    1     1    Mm2
......................................................................ugagaucUugugaaaacugcuu................    1     1    Mm2
......................................................................ugagaucaugugaaaacugcuuG...............    1     1    Mm2
......................................................................ugagaucaugugaaaacugcuuAu..............    19    1    Mm2
......................................................................ugagaucaugugaaaacAgcuuu...............    2     1    Mm2
......................................................................ugagaucaugugaaaacugcuuu...............    451   0    Mm2
......................................................................uAagaucaugugaaaacugcuuu...............    1     1    Mm2
......................................................................ugagaucaugugaaaaAugcuuu...............    3     1    Mm2
......................................................................ugagaucaugugaaaacugUuuu...............    2     1    Mm2
......................................................................ugagaucaugugaaaacugcuuGu..............    1     1    Mm2
......................................................................ugagaucaugugaaaacugcuuCu..............    2     1    Mm2
......................................................................ugagaucaugugaaaacugcuuuA..............    11    1    Mm2
......................................................................Ngagaucaugugaaaacugcuuu...............    1     1    Mm2
......................................................................ugagaucaugugaaaacGgcuuu...............    2     1    Mm2
......................................................................ugagaucaugugaaaaUugcuuu...............    4     1    Mm2
......................................................................ugagaucaugAgaaaacugcuuu...............    1     1    Mm2
......................................................................ugagaucaugugaaaaCCgcuuu...............    1     1    Mm2
......................................................................ugagaucaugugaaaacugcuuuuU.............    26    1    Mm2
.......................................................................gagaucaugugaaaacugcu.................    1     0    Mm2
.......................................................................gagaucauguCaaaacugcuu................    1     1    Mm2
.......................................................................gagaucaugugaaaacugcuu................    2     0    Mm2
.......................................................................gagaucaugugaaaacugcuuu...............    2     0    Mm2
........................................................................agaucaugugaaaacugcuu................    1     0    Mm2
........................................................................agaucaugugaaaacugcuuu...............    1     0    Mm2
.........................................................................gaucaugugaaaacugcuu................    1     0    Mm2

..............................ccgguuuucauaaugauuuuac........................................................    1     0    Mm1
......................................................................ugagaucaugugaaaacA....................    2     1    Mm1
......................................................................ugagaAcaugugaaaacu....................    1     1    Mm1
......................................................................ugagaucaugugaaaacu....................    63    0    Mm1
......................................................................ugagaucaugugaaaacC....................    1     1    Mm1
......................................................................ugagaucaugugaaaacuU...................    12    1    Mm1
......................................................................ugagaucaugugaaaacGg...................    3     1    Mm1
......................................................................ugagaucaugugaaaacug...................    191   0    Mm1
......................................................................ugagaucaugugaGaacug...................    1     1    Mm1
......................................................................ugagaucaugugaaaacCg...................    2     1    Mm1
......................................................................ugagaucaugAgaaaacug...................    1     1    Mm1
......................................................................ugagaucaugugaaaaUug...................    3     1    Mm1
......................................................................ugagaucaugugaaaacuA...................    1     1    Mm1
......................................................................ugagaucaugGgaaaacug...................    1     1    Mm1
......................................................................ugagaucaugugaaaacugU..................    16    1    Mm1
......................................................................ugagaucaugugaaaacugc..................    250   0    Mm1
......................................................................ugagauUaugugaaaacugc..................    1     1    Mm1
......................................................................ugagaucaugugaaaaAugc..................    2     1    Mm1
......................................................................ugagaucaugugaaaacGgc..................    2     1    Mm1
......................................................................ugagCucaugugaaaacugcu.................    1     1    Mm1
......................................................................ugagaucaugugaaaGcugcu.................    1     1    Mm1
......................................................................ugagaucaugugaaaaGugcu.................    1     1    Mm1
......................................................................ugagauGaugugaaaacugcu.................    2     1    Mm1
......................................................................ugagaucaugugaaaCcugcu.................    2     1    Mm1
......................................................................ugagaucaCgugaaaacugcu.................    1     1    Mm1
......................................................................ugagaucaugugaaaacAgcu.................    8     1    Mm1
......................................................................ugagaucaugugaaaacGgcu.................    9     1    Mm1
......................................................................ugagaucaugugaaaacugcu.................    1465  0    Mm1
......................................................................ugagaucaugugaaaaAugcu.................    7     1    Mm1
......................................................................ugagaucaugAgaaaacugcu.................    1     1    Mm1
......................................................................ugagaucaugugaaaaCCgcu.................    5     1    Mm1
......................................................................ugagaucauguCaaaacugcu.................    1     1    Mm1
......................................................................ugagaucaugugaaaacugUu.................    1     1    Mm1
......................................................................ugagGucaugugaaaacugcu.................    1     1    Mm1
......................................................................uUagaucaugugaaaacugcu.................    1     1    Mm1
```

```
                              Star                                    Mature
ggacauggagcucuaccauaaaagacgaaaccgguuuucauaaugauuuuacagauugauuuugaaacucugagaucaugugaaaacugcuuuugucgacagugauca
......................................................................ugagaNcaugugaaaacugcu.................    1      1     Mm1
......................................................................ugagaucaugugaaaaUugcu.................    2      1     Mm1
......................................................................ugagaucaAgugaaaacugcu.................    1      1     Mm1
......................................................................ugagaucaugGgaaaacugcu.................    1      1     Mm1
......................................................................ugagaucaugugaaaaAugcuu................    10     1     Mm1
......................................................................ugagaucaugugaaaacCgcuu................    14     1     Mm1
......................................................................ugagUucaugugaaaacugcuu................    1      1     Mm1
......................................................................ugagaucaugAgaaaacugcuu................    11     1     Mm1
......................................................................ugagaucaugugaaaacAgcuu................    25     1     Mm1
......................................................................ugagaucaugugGaaacugcuu................    1      1     Mm1
......................................................................Ngagaucaugugaaaacugcuu................    1      1     Mm1
......................................................................ugagaucaCgugaaaacugcuu................    2      1     Mm1
......................................................................ugagaucGugugaaaacugcuu................    1      1     Mm1
......................................................................ugagauUaugugaaaacugcuu................    1      1     Mm1
......................................................................ugagaucauguAaaaacugcuu................    1      1     Mm1
......................................................................uCagaucaugugaaaacugcuu................    1      1     Mm1
......................................................................ugagaucaugGgaaaacugcuu................    5      1     Mm1
......................................................................ugagaucaugugaaaacuUcuu................    1      1     Mm1
......................................................................ugagaucaugCgaaaacugcuu................    2      1     Mm1
......................................................................ugagaucaugugaGaacugcuu................    2      1     Mm1
......................................................................ugagaucaugugaaaacGgcuu................    16     1     Mm1
......................................................................ugagaucaugugaaaacugUuu................    13     1     Mm1
......................................................................ugagaucUugugaaaacugcuu................    1      1     Mm1
......................................................................ugagaucaugugaaaaUugcuu................    10     1     Mm1
......................................................................uAagaucaugugaaaacugcuu................    1      1     Mm1
......................................................................ugagaCcaugugaaaacugcuu................    1      1     Mm1
......................................................................ugagaucaugugaaaacugcuu................    3492   0     Mm1
......................................................................ugagaucaugugaaaacuAcuu................    2      1     Mm1
......................................................................ugagaucaugugaCaacugcuu................    1      1     Mm1
......................................................................ugagaucaugugaaaaGugcuu................    3      1     Mm1
......................................................................ugCgaucaugugaaaacugcuu................    1      1     Mm1
......................................................................ugagaucauAugaaaacugcuu................    1      1     Mm1
......................................................................ugaAaucaugugaaaacugcuu................    1      1     Mm1
......................................................................ugUgaucaugugaaaacugcuu................    3      1     Mm1
......................................................................ugaCaucaugugaaaacugcuu................    1      1     Mm1
......................................................................ugagaucauguCaaaacugcuu................    1      1     Mm1
......................................................................ugagaucaugCgaaaacugcuuu...............    1      1     Mm1
......................................................................ugagaNcaugugaaaacugcuuu...............    1      1     Mm1
......................................................................ugagaucaugugaaaacGgcuuu...............    2      1     Mm1
......................................................................Ngagaucaugugaaaacugcuuu...............    1      1     Mm1
......................................................................ugagaucaugugaaaacCgcuuu...............    2      1     Mm1
......................................................................ugagaucaugugaaaacugcuuA...............    23     1     Mm1
......................................................................ugagaucaugugaaaacugcuuu...............    438    0     Mm1
......................................................................ugagaucaugugGaaacugcuuu...............    1      1     Mm1
......................................................................ugagaucaugugaaaacugUuuu...............    4      1     Mm1
......................................................................ugagaucaugAgaaaacugcuuu...............    1      1     Mm1
......................................................................ugagaucaugugaaaaAugcuuu...............    3      1     Mm1
......................................................................ugagaucaugugaaaacAgcuuu...............    1      1     Mm1
......................................................................ugagaucaugugaaaaUugcuuu...............    4      1     Mm1
......................................................................ugagaucaugugaaaacugcuuGu..............    4      1     Mm1
......................................................................ugagaucaugugaaaacugcuAuu..............    4      1     Mm1
......................................................................ugagaucaugugaaaacugcuuuu..............    70     0     Mm1
......................................................................ugGgaucaugugaaaacugcuuuu..............    1      1     Mm1
......................................................................ugagaucaugugaaaacugcuuuuU.............    1      1     Mm1
......................................................................ugagaucaugugaaaacugcuuuuUu............    1      1     Mm1

.....................................................................cugagaucaugugaaaacu....................    1      0     Fm3
.....................................................................cugagaucaugugaaaacugcu.................    7      0     Fm3
.....................................................................cugagaucaugugaaaaGugcuu................    1      1     Fm3
......................................................................ugagaucaugCgaaaacu....................    1      1     Fm3
......................................................................ugaAaucaugugaaaacu....................    1      1     Fm3
......................................................................ugagaucaugugaaaacG....................    10     1     Fm3
......................................................................ugagaucUugugaaaacu....................    1      1     Fm3
......................................................................ugUgaucaugugaaaacu....................    1      1     Fm3
......................................................................ugagaucaugugaaaacu....................    1003   0     Fm3
......................................................................ugagaucaugugaaaaAu....................    7      1     Fm3
......................................................................ugagaucaugugaaaacA....................    6      1     Fm3
......................................................................ugagaucaugugUaaacu....................    1      1     Fm3
......................................................................ugagaucaugAgaaaacu....................    2      1     Fm3
......................................................................ugagaucaugugaaaacC....................    3      1     Fm3
```

```
                                  Star                                         Mature
ggacauggagcucuaccauaaaagacgaaaccgguuuucauaaugauuuuacagauugauuuugaaacucugagaucaugugaaaacugcuuuugucgacagugauca
......................................................................ugagaucaugugGaaacu....................   1      1   Fm3
......................................................................ugaCaucaugugaaaacu....................   1      1   Fm3
......................................................................ugagauAaugugaaaacu....................   1      1   Fm3
......................................................................ugagaucaugugaaaacN....................   1      1   Fm3
......................................................................Cgagaucaugugaaaacu....................   1      1   Fm3
......................................................................uUagaucaugugaaaacu....................   1      1   Fm3
......................................................................ugagaucaugugaaaacAg...................   11     1   Fm3
......................................................................ugagaucaugCgaaaacug...................   5      1   Fm3
......................................................................ugUgaucaugugaaaacug...................   1      1   Fm3
......................................................................ugagaucaugugaaaacGg...................   9      1   Fm3
......................................................................ugagaucaugugaaaacCg...................   12     1   Fm3
......................................................................ugagaucaugugaUaacug...................   1      1   Fm3
......................................................................ugagaucaugugaaaaUug...................   11     1   Fm3
......................................................................Ngagaucaugugaaaacug...................   2      1   Fm3
......................................................................ugagaucaugugaaUacug...................   1      1   Fm3
......................................................................ugagaucaugAgaaaacug...................   3      1   Fm3
......................................................................ugagaucUugugaaaacug...................   1      1   Fm3
......................................................................ugagaucaugugaaaacuA...................   126    1   Fm3
......................................................................ugagaucaugugaaaaAug...................   4      1   Fm3
......................................................................ugagaucaugugaaaacug...................   1635   0   Fm3
......................................................................ugagaucaugGgaaaacug...................   1      1   Fm3
......................................................................ugagaucauUugaaaacug...................   2      1   Fm3
......................................................................ugaAaucaugugaaaacug...................   1      1   Fm3
......................................................................ugagaucauguAaaaacug...................   1      1   Fm3
......................................................................ugGgaucaugugaaaacug...................   1      1   Fm3
......................................................................ugagaucGugugaaaacug...................   1      1   Fm3
......................................................................ugagaucaugugaaaacuU...................   18     1   Fm3
......................................................................ugagaCcaugugaaaacug...................   1      1   Fm3
......................................................................ugagUucaugugaaaacugc..................   1      1   Fm3
......................................................................ugagaucaugugaaaaGugc..................   1      1   Fm3
......................................................................ugagaucaugugaaaacugc..................   1013   0   Fm3
......................................................................ugagauUaugugaaaacugc..................   1      1   Fm3
......................................................................ugGgaucaugugaaaacugc..................   1      1   Fm3
......................................................................ugagaucaugugaaaaAugc..................   6      1   Fm3
......................................................................ugagaucaugugaaaacAgc..................   5      1   Fm3
......................................................................Ngagaucaugugaaaacugc..................   1      1   Fm3
......................................................................ugagaucaugugaaaacGgc..................   5      1   Fm3
......................................................................ugagaucaugAgaaaacugc..................   1      1   Fm3
......................................................................ugagaucaugugaaaacugU..................   24     1   Fm3
......................................................................ugagaucaugugaaaCcugc..................   1      1   Fm3
......................................................................ugagaucaugugaaaacuAc..................   3      1   Fm3
......................................................................uUagaucaugugaaaacugc..................   1      1   Fm3
......................................................................uCagaucaugugaaaacugc..................   1      1   Fm3
......................................................................ugaUaucaugugaaaacugc..................   1      1   Fm3
......................................................................ugagaucaugugaaaaUugc..................   3      1   Fm3
......................................................................ugagaucaugugaaaacCgc..................   4      1   Fm3
......................................................................ugaAaucaugugaaaacugc..................   1      1   Fm3
......................................................................ugagaucauguUaaaacugcu.................   2      1   Fm3
......................................................................Agagaucaugugaaaacugcu.................   1      1   Fm3
......................................................................ugagaucaugugaaaaAugcu.................   10     1   Fm3
......................................................................ugagaucaugugaaaGcugcu.................   1      1   Fm3
......................................................................ugagaucUugugaaaacugcu.................   2      1   Fm3
......................................................................ugagaAcaugugaaaacugcu.................   2      1   Fm3
......................................................................ugagaucaugugaaaaGugcu.................   10     1   Fm3
......................................................................ugagaucauguAaaaacugcu.................   1      1   Fm3
......................................................................ugagaucaugugaaaacGgcu.................   31     1   Fm3
......................................................................ugagaucaugugaGaacugcu.................   2      1   Fm3
......................................................................ugagaucaugugaaaacugcu.................   5772   0   Fm3
......................................................................ugagauUaugugaaaacugcu.................   3      1   Fm3
......................................................................ugagaNcaugugaaaacugcu.................   1      1   Fm3
......................................................................ugagaucaugugaaaacugcC.................   2      1   Fm3
......................................................................ugagaucaugugaaaacugUu.................   5      1   Fm3
......................................................................uCagaucaugugaaaacugcu.................   1      1   Fm3
......................................................................ugagaucaugugaaaUcugcu.................   1      1   Fm3
......................................................................ugagaucaugugaaaacuCcu.................   1      1   Fm3
......................................................................ugagaucaugugaaaacAgcu.................   35     1   Fm3
......................................................................ugagaucaugugaaaaUugcu.................   15     1   Fm3
......................................................................ugagaucauAugaaaacugcu.................   1      1   Fm3
......................................................................ugagaucaugugaaaacugAu.................   1      1   Fm3
......................................................................ugagauAaugugaaaacugcu.................   1      1   Fm3
```

```
                              Star                                       Mature
ggacauggagcucuaccauaaaaagacgaaaccgguuuucauaaugauuuuacagauugauuuugaaacucugagaucaugugaaaacugcuuuugucgacagugauca
.......................................................................Cgagaucaugugaaaacugcu.................   1      1    Fm3
.......................................................................ugagaucaugugaaaacCgcu.................   32     1    Fm3
.......................................................................uAagaucaugugaaaacugcu.................   4      1    Fm3
.......................................................................uUagaucaugugaaaacugcu.................   1      1    Fm3
.......................................................................ugagaucaugAgaaaacugcu.................   10     1    Fm3
.......................................................................ugagaucaugugaaaacuAcu.................   1      1    Fm3
.......................................................................ugagaucaugCgaaaacugcu.................   9      1    Fm3
.......................................................................ugGgaucaugugaaaacugcu.................   4      1    Fm3
.......................................................................Ngagaucaugugaaaacugcu.................   2      1    Fm3
.......................................................................ugagaucaugugaaUacugcu.................   1      1    Fm3
.......................................................................ugagaucaugGgaaaacugcu.................   6      1    Fm3
.......................................................................ugagCucaugugaaaacugcu.................   1      1    Fm3
.......................................................................ugagaucaugugaaaacugcA.................   11     1    Fm3
.......................................................................ugagaucaugugaaaacCgcuu................   50     1    Fm3
.......................................................................Ggagaucaugugaaaacugcuu................   1      1    Fm3
.......................................................................ugagaucaugugaaaacGgcuu................   39     1    Fm3
.......................................................................ugagaucaugugaaGacugcuu................   2      1    Fm3
.......................................................................ugagaucauguUaaaacugcuu................   5      1    Fm3
.......................................................................ugagaucaugugaaaacugcuu................   9949   0    Fm3
.......................................................................ugagaucaugugaaaaUugcuu................   19     1    Fm3
.......................................................................ugagaucaugugaaaacugcCu................   4      1    Fm3
.......................................................................ugagaucaugugaaaacAgcuu................   44     1    Fm3
.......................................................................ugagaCcaugugaaaacugcuu................   1      1    Fm3
.......................................................................ugagaucaugugaaaacuCcuu................   1      1    Fm3
.......................................................................ugagGucaugugaaaacugcuu................   3      1    Fm3
.......................................................................ugagaucauguCaaaacugcuu................   1      1    Fm3
.......................................................................ugagaucaugugaaaacugcuC................   2      1    Fm3
.......................................................................ugagaucaugugUaaacugcuu................   2      1    Fm3
.......................................................................ugGgaucaugugaaaacugcuu................   4      1    Fm3
.......................................................................ugagCucaugugaaaacugcuu................   2      1    Fm3
.......................................................................ugagaucUugugaaaacugcuu................   2      1    Fm3
.......................................................................uAagaucaugugaaaacugcuu................   5      1    Fm3
.......................................................................ugagaucaugugaaaUcugcuu................   1      1    Fm3
.......................................................................ugagaucauguAaaaacugcuu................   2      1    Fm3
.......................................................................ugagaucauCugaaaacugcuu................   1      1    Fm3
.......................................................................ugUgaucaugugaaaacugcuu................   3      1    Fm3
.......................................................................ugagauAaugugaaaacugcuu................   1      1    Fm3
.......................................................................ugagaucaugugaaUacugcuu................   1      1    Fm3
.......................................................................ugagaucaCgugaaaacugcuu................   2      1    Fm3
.......................................................................ugagaucaugugaaaacugGuu................   1      1    Fm3
.......................................................................ugaUaucaugugaaaacugcuu................   1      1    Fm3
.......................................................................ugagaucaugugaaaacugUuu................   25     1    Fm3
.......................................................................ugagaucaugugaaaacuAcuu................   1      1    Fm3
.......................................................................ugagaucaugAgaaaacugcuu................   14     1    Fm3
.......................................................................ugagUucaugugaaaacugcuu................   1      1    Fm3
.......................................................................ugagaucaugugaaaacuUcuu................   1      1    Fm3
.......................................................................Ngagaucaugugaaaacugcuu................   3      1    Fm3
.......................................................................ugagaucaugugaGaacugcuu................   1      1    Fm3
.......................................................................ugagaucaugugaaaaGugcuu................   13     1    Fm3
.......................................................................ugagaucaugCgaaaacugcuu................   9      1    Fm3
.......................................................................ugagaucauUugaaaacugcuu................   1      1    Fm3
.......................................................................ugagaucaugugaaaacugcuA................   9      1    Fm3
.......................................................................ugagaucaugugGaaacugcuu................   1      1    Fm3
.......................................................................Cgagaucaugugaaaacugcuu................   2      1    Fm3
.......................................................................ugaAaucaugugaaaacugcuu................   1      1    Fm3
.......................................................................ugagaucaugugCaaacugcuu................   1      1    Fm3
.......................................................................ugagaucaugugaaaCcugcuu................   1      1    Fm3
.......................................................................ugagauUaugugaaaacugcuu................   1      1    Fm3
.......................................................................ugagaucaugugaaaaAugcuu................   42     1    Fm3
.......................................................................ugagaucauAugaaaacugcuu................   2      1    Fm3
.......................................................................ugaCaucaugugaaaacugcuu................   2      1    Fm3
.......................................................................uUagaucaugugaaaacugcuu................   5      1    Fm3
.......................................................................uCagaucaugugaaaacugcuu................   2      1    Fm3
.......................................................................ugagaucaugGgaaaacugcuu................   8      1    Fm3
.......................................................................ugagaucaugugaaaacAgcuuu...............   3      1    Fm3
.......................................................................ugagaucaugugaaaacugcGuu...............   1      1    Fm3
.......................................................................ugagaucauguUaaaacugcuuu...............   1      1    Fm3
.......................................................................ugGgaucaugugaaaacugcuuu...............   1      1    Fm3
.......................................................................ugagaucaugugaaaacugcuuA...............   65     1    Fm3
.......................................................................ugagaucaugugaaaaAugcuuu...............   2      1    Fm3
```

```
                              Star                                    Mature
ggacauggagcucuaccauaaaagacgaaaccgguuuucauaaugauuuuacagauugauuuugaaacucugagaucaugugaaaacugcuuuugucgacagugauca
......................................................................ugagaucaugugaaaCcugcuuu................   1      1      Fm3
......................................................................ugagaucaugCgaaaacugcuuu................   1      1      Fm3
......................................................................ugagaucaugugaaaacugcuAu................   1      1      Fm3
......................................................................ugagaucaugugaaaaUugcuuu................   1      1      Fm3
......................................................................ugagaucaugugaaaacGgcuuu................   4      1      Fm3
......................................................................ugagaucaugugaaaacugUuuu................   7      1      Fm3
......................................................................ugagaucUugugaaaacugcuuu................   1      1      Fm3
......................................................................ugagaucaugugaaaacugcuuG................   3      1      Fm3
......................................................................ugagaucaugugaaaaGugcuuu................   1      1      Fm3
......................................................................ugagaucaugugaaaUcugcuuu................   1      1      Fm3
......................................................................ugagaucaugugaaaacCgcuuu................   1      1      Fm3
......................................................................ugagaucaugugaaaacugcuuu................   915    0      Fm3
......................................................................ugagaucaugAgaaaacugcuuu................   1      1      Fm3
......................................................................Cgagaucaugugaaaacugcuuu................   1      1      Fm3
......................................................................ugagaucaugugaaaacugcuuuA...............   2      1      Fm3
......................................................................ugagaucaugugaaaacugUuuuu...............   1      1      Fm3
......................................................................ugagaucaugugaaaaUugcuuuu...............   1      1      Fm3
......................................................................ugagaucaugugaaaacugcuuuG...............   2      1      Fm3
......................................................................ugagaucaugugaaaacugcuuAu...............   8      1      Fm3
......................................................................ugagaucaugugaaaacugcuuuu...............   100    0      Fm3
......................................................................ugagaucaugugaaaacugcuuGu...............   1      1      Fm3
......................................................................ugagaucaugAgaaaacugcuuuu...............   1      1      Fm3
......................................................................ugagaucaugugaaaacAgcuuuu...............   1      1      Fm3
......................................................................ugagaucaugugaaaacugcuuuuU..............   3      1      Fm3
......................................................................ugagaucaugugaaaacugcuuuuA..............   1      1      Fm3
.......................................................................gagaucaugugaaaacugcu..................   4      0      Fm3
.......................................................................gagaucaugugaaaacugcuu.................   3      0      Fm3
.......................................................................gagaucauUugaaaacugcuu.................   1      1      Fm3
.......................................................................Uagaucaugugaaaacugcuu.................   1      1      Fm3
.......................................................................gagaucaugugaaaacugcuuu................   1      0      Fm3
.......................................................................gagaucaugugaaaacugcuuuu...............   1      0      Fm3
........................................................................Ugaucaugugaaaacugcuu.................   2      1      Fm3

..............................ccgguuuucauaaugauu............................................................   6      0      Fm2
..............................ccgguuuucauaaugauuuuac........................................................   38     0      Fm2
..............................ccgguuuucauaaugauAuuaca.......................................................   1      1      Fm2
..............................ccgguuuucauaaugauuuuUca.......................................................   1      1      Fm2
..............................ccgguuuucauaaugUuuuuaca.......................................................   1      1      Fm2
..............................ccgguuuucauaaugauuuuaca.......................................................   132    0      Fm2
..............................ccgguuuucauaaugauuuuacagG.....................................................   1      1      Fm2
..............................ccgguuuucauaaugauGuuacaga.....................................................   1      1      Fm2
..............................ccgguuuucauaaugauCuuacaga.....................................................   1      1      Fm2
..............................ccgguuuucauaaugauuuuacaga.....................................................   66     0      Fm2
..............................ccgguuuucauaaugaAuuuacaga.....................................................   1      1      Fm2
..............................ccgguuuucauaaugaGuuuacaga.....................................................   1      1      Fm2
.....................................................................cugagaucaugugaaaacugcG.................   1      1      Fm2
.....................................................................cugagaucaugugaaaacugcu.................   32     0      Fm2
......................................................................Ngagaucaugugaaaacu....................   1      1      Fm2
......................................................................ugagaucaugugaaaacG....................   1      1      Fm2
......................................................................ugagaucaugugaaaacA....................   1      1      Fm2
......................................................................ugagaucaugugaaaacu....................   204    0      Fm2
......................................................................ugagaucaugugaaaacug...................   2387   0      Fm2
......................................................................ugagaucaugCgaaaacug...................   4      1      Fm2
......................................................................ugagaucaugugUaaacug...................   1      1      Fm2
......................................................................ugagaucaugugaaaacGg...................   31     1      Fm2
......................................................................uUagaucaugugaaaacug...................   1      1      Fm2
......................................................................Ngagaucaugugaaaacug...................   1      1      Fm2
......................................................................ugagaucaugugaGaacug...................   1      1      Fm2
......................................................................ugagUucaugugaaaacug...................   1      1      Fm2
......................................................................ugagaucauguAaaaacug...................   1      1      Fm2
......................................................................ugagaucaugugaaaaAug...................   11     1      Fm2
......................................................................uAagaucaugugaaaacug...................   2      1      Fm2
......................................................................ugagaucaugugaaaacAg...................   17     1      Fm2
......................................................................ugagaAcaugugaaaacug...................   1      1      Fm2
......................................................................Cgagaucaugugaaaacug...................   1      1      Fm2
......................................................................ugaAaucaugugaaaacug...................   1      1      Fm2
......................................................................ugagaucaugugaaaacuU...................   16     1      Fm2
......................................................................ugagaucaugugaaaacCg...................   8      1      Fm2
......................................................................ugagaucaugugaaaacuA...................   11     1      Fm2
......................................................................ugagaucauguUaaaacug...................   1      1      Fm2
```

Star Mature

ggacauggagcucuaccauaaaagacgaaaccgguuuucauaaugauuuuacagauugauuuugaaacucugagaucaugugaaaacugcuuuugucgacagugauca			
....ugagaucaugugaaaaUug...................	3	1	Fm2
....ugagaucaugAgaaaacug...................	6	1	Fm2
....ugagGucaugugaaaacug...................	1	1	Fm2
....ugagaucaugugaaCacug...................	1	1	Fm2
....ugagaucaugGgaaaacug...................	1	1	Fm2
....uUagaucaugugaaaacugc..................	2	1	Fm2
....ugagaucaugugGaaacugc..................	1	1	Fm2
....ugagaucaugugaaaUcugc..................	2	1	Fm2
....ugagaucaugugaaaaAugc..................	9	1	Fm2
....ugagaucaugugaaaacCgc..................	8	1	Fm2
....ugagaCcaugugaaaacugc..................	1	1	Fm2
....ugagaucaugGgaaaacugc..................	1	1	Fm2
....ugagaucaugugaaaaUugc..................	2	1	Fm2
....ugagaucaugugaaaacugc..................	1846	0	Fm2
....ugagaucaugugaaaacuUc..................	1	1	Fm2
....ugagaucaugugaaaacugU..................	12	1	Fm2
....ugagaucaugugaUaacugc..................	1	1	Fm2
....ugagaucaugAgaaaacugc..................	8	1	Fm2
....ugagaucaugugaaaacAgc..................	14	1	Fm2
....ugagGucaugugaaaacugc..................	1	1	Fm2
....ugagaucaugugaaaacugG..................	1	1	Fm2
....ugUgaucaugugaaaacugc..................	1	1	Fm2
....ugagaucaugCgaaaacugc..................	2	1	Fm2
....ugagaucaugugaaaaGugc..................	4	1	Fm2
....ugagaucUugugaaaacugc..................	1	1	Fm2
....ugagaucaugugaaaacGgc..................	15	1	Fm2
....ugagaucaugugaCaacugcu.................	2	1	Fm2
....ugagaucaugugaaaCcugcu.................	2	1	Fm2
....ugagaAcaugugaaaacugcu.................	5	1	Fm2
....ugagaucaAgugaaaacugcu.................	1	1	Fm2
....ugagUucaugugaaaacugcu.................	3	1	Fm2
....ugagaucaugugaaaacGgcu.................	75	1	Fm2
....ugagaCcaugugaaaacugcu.................	3	1	Fm2
....ugagaucaugugaaaacugcu.................	14644	0	Fm2
....uAagaucaugugaaaacugcu.................	6	1	Fm2
....ugagauAaugugaaaacugcu.................	3	1	Fm2
....ugagaucUugugaaaacugcu.................	2	1	Fm2
....ugagaucaugugaaaacuCcu.................	1	1	Fm2
....ugagaucaugugaaaacCgcu.................	75	1	Fm2
....ugagaucaugugaaaacAgcu.................	63	1	Fm2
....ugagaucauguUaaaacugcu.................	1	1	Fm2
....uUagaucaugugaaaacugcu.................	1	1	Fm2
....ugagaucaugugaaaacugcA.................	34	1	Fm2
....ugagGucaugugaaaacugcu.................	5	1	Fm2
....ugagaucaugugaaaacuAcu.................	1	1	Fm2
....ugagaucauguCaaaacugcu.................	2	1	Fm2
....ugagaucaugugaaaGcugcu.................	3	1	Fm2
....ugagaucauAugaaaacugcu.................	3	1	Fm2
....ugagaucaugCgaaaacugcu.................	17	1	Fm2
....ugagaucaugugaGaacugcu.................	3	1	Fm2
....ugGgaucaugugaaaacugcu.................	2	1	Fm2
....uCagaucaugugaaaacugcu.................	2	1	Fm2
....ugaCaucaugugaaaacugcu.................	3	1	Fm2
....ugagaucaugugaaaacuUcu.................	1	1	Fm2
....ugagCucaugugaaaacugcu.................	3	1	Fm2
....ugagaucauguAaaaacugcu.................	1	1	Fm2
....ugagaucaugugaaaacugUu.................	7	1	Fm2
....ugUgaucaugugaaaacugcu.................	2	1	Fm2
....ugagaucaugugaaaacugAu.................	2	1	Fm2
....ugagaNcaugugaaaacugcu.................	2	1	Fm2
....ugagaucaugugUaaacugcu.................	2	1	Fm2
....ugagaucaCgugaaaacugcu.................	6	1	Fm2
....ugagauUaugugaaaacugcu.................	7	1	Fm2
....ugagaucaugAgaaaacugcu.................	27	1	Fm2
....ugagaucaugugaaaacugcC.................	1	1	Fm2
....ugagaucaugugaUaacugcu.................	1	1	Fm2
....ugagaucaugugGaaacugcu.................	4	1	Fm2
....Ngagaucaugugaaaacugcu.................	5	1	Fm2
....Agagaucaugugaaaacugcu.................	1	1	Fm2
....ugagaucaugugaaaaUugcu.................	32	1	Fm2

Star / Mature			
ggacauggagcucuaccauaaaagacgaaaccgguuuucauaaugauuuuacagauugauuuugaaacucugagaucaugugaaaacugcuuuugucgacagugauca			
....ugagaucaugGgaaaacugcu.................	13	1	Fm2
....ugagaucCugugaaaacugcu.................	1	1	Fm2
....Cgagaucaugugaaaacugcu.................	2	1	Fm2
....ugagaucaugugaaaaGugcu.................	23	1	Fm2
....ugagaucaugugaaaaAugcu.................	68	1	Fm2
....ugagaucaugugaaaacugcG.................	49	1	Fm2
....ugagaucGugugaaaacugcu.................	1	1	Fm2
....ugagaucaugugaaaUcugcu.................	3	1	Fm2
....Ngagaucaugugaaaacugcuu................	10	1	Fm2
....ugagUucaugugaaaacugcuu................	1	1	Fm2
....Cgagaucaugugaaaacugcuu................	6	1	Fm2
....ugagauNaugugaaaacugcuu................	3	1	Fm2
....ugagaucaugugaaaGcugcuu................	2	1	Fm2
....ugagaucaugugaaaacugcuu................	26527	0	Fm2
....ugagaucaCgugaaaacugcuu................	4	1	Fm2
....ugagaucauUugaaaacugcuu................	2	1	Fm2
....ugagaucaugugaCaacugcuu................	2	1	Fm2
....ugaCaucaugugaaaacugcuu................	1	1	Fm2
....ugagaucauguCaaaacugcuu................	2	1	Fm2
....ugagaucaugugCaaacugcuu................	3	1	Fm2
....ugagaucauguUaaaacugcuu................	2	1	Fm2
....ugagauUaugugaaaacugcuu................	10	1	Fm2
....ugagaucGugugaaaacugcuu................	3	1	Fm2
....ugagaucaugugaaaaUugcuu................	44	1	Fm2
....ugagaucaugugaaaUcugcuu................	5	1	Fm2
....ugagaucaugugUaaacugcuu................	6	1	Fm2
....ugagaucaugugaaUacugcuu................	4	1	Fm2
....ugagauGaugugaaaacugcuu................	2	1	Fm2
....ugagaucauAugaaaacugcuu................	2	1	Fm2
....ugagaucauguAaaaacugcuu................	2	1	Fm2
....ugagaCcaugugaaaacugcuu................	1	1	Fm2
....Agagaucaugugaaaacugcuu................	3	1	Fm2
....ugagaucaugCgaaaacugcuu................	28	1	Fm2
....ugGgaucaugugaaaacugcuu................	22	1	Fm2
....ugagaAcaugugaaaacugcuu................	2	1	Fm2
....ugagaucaAgugaaaacugcuu................	2	1	Fm2
....ugUgaucaugugaaaacugcuu................	5	1	Fm2
....ugagaucaugugaaaaGugcuu................	37	1	Fm2
....ugagaNcaugugaaaacugcuu................	3	1	Fm2
....ugagaucaugugaaaacuUcuu................	2	1	Fm2
....ugagaucaugugaaaacugcuG................	22	1	Fm2
....ugagaucaugugaUaacugcuu................	2	1	Fm2
....uUagaucaugugaaaacugcuu................	3	1	Fm2
....ugagaucaugugaaaacugcuC................	2	1	Fm2
....ugagaucaugugaaaacugcCu................	2	1	Fm2
....ugagauAaugugaaaacugcuu................	3	1	Fm2
....ugagCucaugugaaaacugcuu................	10	1	Fm2
....ugagaucaugugaaaacugUuu................	36	1	Fm2
....ugagaucaugugaaaCcugcuu................	2	1	Fm2
....ugaUaucaugugaaaacugcuu................	1	1	Fm2
....ugagGucaugugaaaacugcuu................	5	1	Fm2
....ugagaucaugugaaaacGgcuu................	124	1	Fm2
....ugagaucaugugGaaacugcuu................	4	1	Fm2
....ugagaucaugugaaaacAgcuu................	149	1	Fm2
....ugagaucUugugaaaacugcuu................	7	1	Fm2
....ugagaucaugugaGaacugcuu................	1	1	Fm2
....ugNgaucaugugaaaacugcuu................	1	1	Fm2
....ugagaucaugugaaGacugcuu................	6	1	Fm2
....ugagaucaugugaaaacCgcuu................	153	1	Fm2
....ugagaucaugugaaaacugcuA................	30	1	Fm2
....ugagaucaugugaaaaAugcuu................	89	1	Fm2
....ugagaucaugugaaaacuAcuu................	3	1	Fm2
....uCagaucaugugaaaacugcuu................	1	1	Fm2
....ugagaucaugugaaaacugAuu................	3	1	Fm2
....ugagaucaugAgaaaacugcuu................	46	1	Fm2
....uAagaucaugugaaaacugcuu................	9	1	Fm2
....ugagaucauCugaaaacugcuu................	3	1	Fm2
....ugaAaucaugugaaaacugcuu................	3	1	Fm2
....uNagaucaugugaaaacugcuu................	1	1	Fm2
....ugagaucaugGgaaaacugcuu................	29	1	Fm2

```
                              Star                                    Mature
ggacauggagcucuaccauaaaagacgaaaccgguuuucauaaugauuuuacagauugauuuugaaacucugagaucaugugaaaacugcuuuugucgacagugauca
......................................................................ugagaucaugugaaaacugcGu................   1      1      Fm2
......................................................................ugagaucaugugaaaacGgcuuu...............   12     1      Fm2
......................................................................ugagaucaugugaaaacugcuAu...............   1      1      Fm2
......................................................................ugagaucauguUaaaacugcuuu...............   1      1      Fm2
......................................................................ugCgaucaugugaaaacugcuuu...............   1      1      Fm2
......................................................................ugagaucaugugaaaaGugcuuu...............   5      1      Fm2
......................................................................ugagaucaugugaaaacugcuuu...............   3250   0      Fm2
......................................................................ugagaucGugugaaaacugcuuu...............   1      1      Fm2
......................................................................ugagaucaugugaaaacugcuuC...............   22     1      Fm2
......................................................................ugagCucaugugaaaacugcuuu...............   1      1      Fm2
......................................................................ugUgaucaugugaaaacugcuuu...............   1      1      Fm2
......................................................................ugagaucaugugUaaacugcuuu...............   1      1      Fm2
......................................................................ugagaucaugugaaaacugcuuG...............   22     1      Fm2
......................................................................ugagaucaugAgaaaacugcuuu...............   3      1      Fm2
......................................................................ugagaucaugugaaaacugcuuA...............   250    1      Fm2
......................................................................ugaAaucaugugaaaacugcuuu...............   1      1      Fm2
......................................................................ugagaucaugGgaaaacugcuuu...............   7      1      Fm2
......................................................................ugagaucaugugaaaacAgcuuu...............   22     1      Fm2
......................................................................uAagaucaugugaaaacugcuuu...............   3      1      Fm2
......................................................................ugagaucaugugaaaacCgcuuu...............   23     1      Fm2
......................................................................Ngagaucaugugaaaacugcuuu...............   2      1      Fm2
......................................................................ugagaucaugugaaaaAugcuuu...............   16     1      Fm2
......................................................................ugagaucCugugaaaacugcuuu...............   1      1      Fm2
......................................................................ugagaucaugugaaaaUugcuuu...............   6      1      Fm2
......................................................................ugGgaucaugugaaaacugcuuu...............   4      1      Fm2
......................................................................ugagaucaugCgaaaacugcuuu...............   5      1      Fm2
......................................................................Cgagaucaugugaaaacugcuuu...............   2      1      Fm2
......................................................................ugagaucaugugaaaacugcCuu...............   1      1      Fm2
......................................................................ugaUaucaugugaaaacugcuuu...............   1      1      Fm2
......................................................................ugagUucaugugaaaacugcuuu...............   1      1      Fm2
......................................................................ugagaucauguAaaaacugcuuu...............   1      1      Fm2
......................................................................ugagaucaugugaaaacugUuuu...............   7      1      Fm2
......................................................................ugagaucaugugaaaacCgcuuuu..............   5      1      Fm2
......................................................................ugagaucaugugaaaacGgcuuuu..............   7      1      Fm2
......................................................................ugagCucaugugaaaacugcuuuu..............   2      1      Fm2
......................................................................Agagaucaugugaaaacugcuuuu..............   1      1      Fm2
......................................................................ugagaucaugugaaaaUugcuuuu..............   5      1      Fm2
......................................................................ugagaucaugugaaaacugcuuuu..............   1101   0      Fm2
......................................................................ugGgaucaugugaaaacugcuuuu..............   1      1      Fm2
......................................................................ugagaNcaugugaaaacugcuuuu..............   1      1      Fm2
......................................................................ugagaucaugugaaaacAgcuuuu..............   3      1      Fm2
......................................................................ugagaucaugugaaaacugcCuuu..............   1      1      Fm2
......................................................................ugagaucaugugaaaacugcuuuA..............   22     1      Fm2
......................................................................ugagaucaugugaaaaAugcuuuu..............   6      1      Fm2
......................................................................uAagaucaugugaaaacugcuuuu..............   1      1      Fm2
......................................................................ugagaucaugugaaaacugcuuGu..............   41     1      Fm2
......................................................................ugagaucaugAgaaaacugcuuuu..............   2      1      Fm2
......................................................................ugagaucUugugaaaacugcuuuu..............   1      1      Fm2
......................................................................ugagaucaugugaaaacugcuuAu..............   46     1      Fm2
......................................................................ugagaucaugugaaaacugcuuuuU.............   69     1      Fm2
.......................................................................gagaucaugugaaaacugcuu................   4      0      Fm2
............................................................................caugugaaaacugcuuuugucgaca.......   13     0      Fm2

..............................ccgguuuucaCaaugauu............................................................   2      1      Fm1
..............................ccgguuuucauaaugauuuu..........................................................   4      0      Fm1
..............................ccgguuuucauaaugauGuuac........................................................   1      1      Fm1
..............................ccgguuuucauaaugauuuuac........................................................   5      0      Fm1
..............................ccgguuuucaCaaugauuuuac........................................................   12     1      Fm1
..............................Ucgguuuucauaaugauuuuaca.......................................................   1      1      Fm1
..............................ccgguuuucauUaugauuuuaca.......................................................   1      1      Fm1
..............................ccgguuuucauaaugauCuuaca.......................................................   1      1      Fm1
..............................ccgguuuucaCaaugauuuuaca.......................................................   7      1      Fm1
..............................ccgguuuucauaaugauuuuaca.......................................................   25     0      Fm1
..............................ccgguuuucauaaugauuuuacaga.....................................................   19     0      Fm1
..............................ccgguuuucauaaugauCuuacaga.....................................................   1      1      Fm1
..............................ccgguuuucaCaaugauuuuacaga.....................................................   17     1      Fm1
....................................................................ucugagaucaugugaaaacu....................   2      0      Fm1
....................................................................ucugagaucaugugaaaacugc..................   3      0      Fm1
.....................................................................cugagaucaugugaaaaAugcu.................   1      1      Fm1
.....................................................................cugagaucaugugaaaacugcu.................   12     0      Fm1
```

Star Mature

```
ggacauggagcucuaccauaaaagacgaaaccgguuuucauaaugauuuuacagauugauuuugaaacucugagaucaugugaaaacugcuuuugucgacagugauca
.....................................................................cugagaucaugugaaaacugcuu................  9     0  Fm1
......................................................................ugagaucaugugaaaaUu....................  1     1  Fm1
......................................................................ugagaucaugugaaaacA....................  3     1  Fm1
......................................................................ugagaucaugugaaaacu....................  328   0  Fm1
......................................................................ugagaucaugugaaaacC....................  1     1  Fm1
......................................................................ugagaucaugugaGaacu....................  1     1  Fm1
......................................................................ugagaucaugugaaUacu....................  1     1  Fm1
......................................................................ugagaucaugugaaaaAu....................  1     1  Fm1
......................................................................ugagaucauUugaaaacu....................  1     1  Fm1
......................................................................ugagaucaugugaaGacu....................  1     1  Fm1
......................................................................ugagaucaugugaaaacG....................  2     1  Fm1
......................................................................ugaCaucaugugaaaacug...................  1     1  Fm1
......................................................................ugGgaucaugugaaaacug...................  1     1  Fm1
......................................................................ugagaucaugugaaaaAug...................  5     1  Fm1
......................................................................ugagaucaugugaaaacuU...................  15    1  Fm1
......................................................................ugagaucaugAgaaaacug...................  3     1  Fm1
......................................................................ugagaucaugugaaaacCg...................  12    1  Fm1
......................................................................ugagaucaugugaCaacug...................  1     1  Fm1
......................................................................ugagaucaugugaaaacAg...................  5     1  Fm1
......................................................................ugagaucaugugaaaaUug...................  3     1  Fm1
......................................................................ugagaucauCugaaaacug...................  1     1  Fm1
......................................................................ugagaucaugugaaaacuA...................  40    1  Fm1
......................................................................ugagaucaugugaaaacug...................  1196  0  Fm1
......................................................................ugagaucaugugaaaacGg...................  12    1  Fm1
......................................................................ugagaucaugugaaaacuC...................  1     1  Fm1
......................................................................ugagaucauUugaaaacug...................  1     1  Fm1
......................................................................ugagaucaugugaaaacugG..................  2     1  Fm1
......................................................................ugagaucaugugaaaacAgc..................  9     1  Fm1
......................................................................ugagaucaugugaaaaAugc..................  6     1  Fm1
......................................................................ugagaucaugugGaaacugc..................  1     1  Fm1
......................................................................ugagaucaugugaaaacCgc..................  6     1  Fm1
......................................................................ugagaucaugugaaaacGgc..................  8     1  Fm1
......................................................................ugagaucaugugaaaacugU..................  41    1  Fm1
......................................................................ugagaucaugugaaaaGugc..................  2     1  Fm1
......................................................................ugagaucaugGgaaaacugc..................  1     1  Fm1
......................................................................Ggagaucaugugaaaacugc..................  1     1  Fm1
......................................................................ugagaucaugugCaaacugc..................  1     1  Fm1
......................................................................ugagaucaugugaaaaUugc..................  1     1  Fm1
......................................................................ugagaucaugugaaaacugc..................  1117  0  Fm1
......................................................................ugagaucaugAgaaaacugc..................  1     1  Fm1
......................................................................ugagaucaugugaaaacugcC.................  2     1  Fm1
......................................................................ugCgaucaugugaaaacugcu.................  1     1  Fm1
......................................................................ugGgaucaugugaaaacugcu.................  2     1  Fm1
......................................................................ugagaucaugGgaaaacugcu.................  3     1  Fm1
......................................................................ugagaNcaugugaaaacugcu.................  1     1  Fm1
......................................................................ugagaucaugugaaaaAugcu.................  15    1  Fm1
......................................................................ugUgaucaugugaaaacugcu.................  2     1  Fm1
......................................................................ugaAaucaugugaaaacugcu.................  1     1  Fm1
......................................................................ugagaucaugugaaaacugcG.................  12    1  Fm1
......................................................................ugagaucaCgugaaaacugcu.................  2     1  Fm1
......................................................................ugagaucauAugaaaacugcu.................  1     1  Fm1
......................................................................ugagaucaugugaaaacuAcu.................  1     1  Fm1
......................................................................Ngagaucaugugaaaacugcu.................  2     1  Fm1
......................................................................ugagaucauUugaaaacugcu.................  1     1  Fm1
......................................................................ugagaucaugugaaaacuUcu.................  1     1  Fm1
......................................................................ugagaucaugugaaaacCgcu.................  24    1  Fm1
......................................................................ugagaucaugugaaaaUugcu.................  9     1  Fm1
......................................................................ugagaucaugugaaGacugcu.................  1     1  Fm1
......................................................................ugagaucaugugaaaaGugcu.................  6     1  Fm1
......................................................................ugagaucaugugaaaacGgcu.................  18    1  Fm1
......................................................................ugagaGcaugugaaaacugcu.................  1     1  Fm1
......................................................................Cgagaucaugugaaaacugcu.................  1     1  Fm1
......................................................................ugagaucaugugaaaacugcu.................  3519  0  Fm1
......................................................................Ggagaucaugugaaaacugcu.................  1     1  Fm1
......................................................................ugagaucaugugaaaacAgcu.................  11    1  Fm1
......................................................................ugagaucaugugGaaacugcu.................  2     1  Fm1
......................................................................ugagaucaugAgaaaacugcu.................  7     1  Fm1
......................................................................ugagCucaugugaaaacugcu.................  2     1  Fm1
......................................................................ugagauAaugugaaaacugcu.................  1     1  Fm1
......................................................................ugaguucauguAaaaacugcu.................  1     1  Fm1
```

```
                                  Star                                         Mature
ggacauggagcucuaccauaaaagacgaaaccgguuuucauaaugauuuuacagauugauuuugaaacucugagaucaugugaaaacugcuuuugucgacagugauca
......................................................................ugagaucaugugaCaacugcu.................  1      1   Fm1
......................................................................ugagaucaugugaaUacugcu.................  1      1   Fm1
......................................................................ugagaucaugugUaaacugcu.................  1      1   Fm1
......................................................................ugagaucaugugaaaacugcA.................  9      1   Fm1
......................................................................ugagaucaugugaaaacugUu.................  16     1   Fm1
......................................................................uAagaucaugugaaaacugcu.................  2      1   Fm1
......................................................................ugagaucaugugaaaacugAu.................  2      1   Fm1
......................................................................ugagaucaugCgaaaacugcu.................  7      1   Fm1
......................................................................ugagaucaAgugaaaacugcuu................  1      1   Fm1
......................................................................uCagaucaugugaaaacugcuu................  3      1   Fm1
......................................................................ugagaucaugugaaaacuAcuu................  3      1   Fm1
......................................................................ugagauAaugugaaaacugcuu................  1      1   Fm1
......................................................................ugagaucaugugaaaacugcuA................  3      1   Fm1
......................................................................ugagauNaugugaaaacugcuu................  2      1   Fm1
......................................................................ugagaucaugugaaaacugGuu................  2      1   Fm1
......................................................................ugagaucaugugaaaacugcCu................  5      1   Fm1
......................................................................ugagaucaugugaaGacugcuu................  1      1   Fm1
......................................................................ugagaucaugGgaaaacugcuu................  16     1   Fm1
......................................................................ugagaucaugCgaaaacugcuu................  14     1   Fm1
......................................................................ugagaucUugugaaaacugcuu................  7      1   Fm1
......................................................................Ggagaucaugugaaaacugcuu................  1      1   Fm1
......................................................................ugagCucaugugaaaacugcuu................  8      1   Fm1
......................................................................ugagaucauAugaaaacugcuu................  2      1   Fm1
......................................................................ugagaCcaugugaaaacugcuu................  2      1   Fm1
......................................................................ugagaucaugugGaaacugcuu................  1      1   Fm1
......................................................................ugagaucaugugaaaaGugcuu................  32     1   Fm1
......................................................................ugagaucaugAgaaaacugcuu................  31     1   Fm1
......................................................................ugagaucauguUaaaacugcuu................  1      1   Fm1
......................................................................ugUgaucaugugaaaacugcuu................  2      1   Fm1
......................................................................ugagaucaugugaGaacugcuu................  4      1   Fm1
......................................................................ugagauGaugugaaaacugcuu................  3      1   Fm1
......................................................................ugagaucaugugaaaacugAuu................  6      1   Fm1
......................................................................ugagaucaugugaUaacugcuu................  2      1   Fm1
......................................................................ugaCaucaugugaaaacugcuu................  2      1   Fm1
......................................................................ugGgaucaugugaaaacugcuu................  9      1   Fm1
......................................................................ugCgaucaugugaaaacugcuu................  1      1   Fm1
......................................................................ugagaucaGgugaaaacugcuu................  1      1   Fm1
......................................................................ugagaucaugugaaaacAgcuu................  85     1   Fm1
......................................................................ugagaucaCgugaaaacugcuu................  3      1   Fm1
......................................................................ugagaucaugugaaaacugcGu................  3      1   Fm1
......................................................................ugagaucaugugUaaacugcuu................  1      1   Fm1
......................................................................ugagaucauUugaaaacugcuu................  1      1   Fm1
......................................................................ugagauUaugugaaaacugcuu................  7      1   Fm1
......................................................................ugagaucaugugaaaacugUuu................  63     1   Fm1
......................................................................ugagaNcaugugaaaacugcuu................  2      1   Fm1
......................................................................ugagaucaugugaaaaAugcuu................  71     1   Fm1
......................................................................ugagaucaugugaaaacugcuC................  2      1   Fm1
......................................................................ugagaucaugugaaaacugcuG................  7      1   Fm1
......................................................................ugagaucaugugaaCacugcuu................  1      1   Fm1
......................................................................ugagaucauCugaaaacugcuu................  2      1   Fm1
......................................................................Agagaucaugugaaaacugcuu................  4      1   Fm1
......................................................................ugaAaucaugugaaaacugcuu................  1      1   Fm1
......................................................................ugagaucaugugaaaaUugcuu................  38     1   Fm1
......................................................................ugagaucaugugaaaacugcuu................  17152  0   Fm1
......................................................................uAagaucaugugaaaacugcuu................  9      1   Fm1
......................................................................Ngagaucaugugaaaacugcuu................  9      1   Fm1
......................................................................ugagaucaugugaCaacugcuu................  4      1   Fm1
......................................................................ugagGucaugugaaaacugcuu................  3      1   Fm1
......................................................................ugagaucaugugaaaUcugcuu................  3      1   Fm1
......................................................................Cgagaucaugugaaaacugcuu................  5      1   Fm1
......................................................................ugagaucaugugaaaaCCgcuu................  97     1   Fm1
......................................................................ugagaucGugugaaaacugcuu................  3      1   Fm1
......................................................................ugagUucaugugaaaacugcuu................  1      1   Fm1
......................................................................uUagaucaugugaaaacugcuu................  5      1   Fm1
......................................................................ugagaucaugugaaaacGgcuu................  76     1   Fm1
......................................................................ugagaucaugugCaaacugcuu................  2      1   Fm1
......................................................................ugagaucaugugaaaacugcuuu...............  1835   0   Fm1
......................................................................ugCgaucaugugaaaacugcuuu...............  1      1   Fm1
......................................................................ugGgaucaugugaaaacugcuuu...............  1      1   Fm1
......................................................................ugagaucaugAgaaaacugcuuu...............  7      1   Fm1
```

```
                             Star                                        Mature
ggacauggagcucuaccauaaaagacgaaaccgguuuucauaaugauuuuacagauugauuuugaaacucugagaucaugugaaaacugcuuuugucgacagugauca
......................................................................ugagaucaugugaaaacCgcuuu...............   13    1    Fm1
......................................................................ugagaucaugugaaaacugcuCu...............   1     1    Fm1
......................................................................ugaUaucaugugaaaacugcuuu...............   1     1    Fm1
......................................................................ugagaucaugugaaaacugcuuA...............   92    1    Fm1
......................................................................ugagaucaugugaaaacGgcuuu...............   5     1    Fm1
......................................................................ugagaucaugugaaaacAgcuuu...............   9     1    Fm1
......................................................................ugagaucaugugaaaaGugcuuu...............   3     1    Fm1
......................................................................ugagaucaugugaGaacugcuuu...............   1     1    Fm1
......................................................................ugagaucaugugaaaacugcuuG...............   6     1    Fm1
......................................................................ugagaucaugugaaaacugUuuu...............   8     1    Fm1
......................................................................ugagaucaugugaaaacuUcuuu...............   1     1    Fm1
......................................................................ugagaucaugugCaaacugcuuu...............   1     1    Fm1
......................................................................ugagaucaugugaaaacugcuuC...............   5     1    Fm1
......................................................................ugagauAaugugaaaacugcuuu...............   1     1    Fm1
......................................................................ugagaucaugugaaaaUugcuuu...............   7     1    Fm1
......................................................................ugagaucaugugaaaaAugcuuu...............   2     1    Fm1
......................................................................uAagaucaugugaaaacugcuuu...............   1     1    Fm1
......................................................................ugagauUaugugaaaacugcuuu...............   2     1    Fm1
......................................................................Cgagaucaugugaaaacugcuuu...............   2     1    Fm1
......................................................................Ngagaucaugugaaaacugcuuu...............   2     1    Fm1
......................................................................ugagaucaugugaaaacugcAuu...............   1     1    Fm1
......................................................................ugagaucaugugaaaacugcCuu...............   1     1    Fm1
......................................................................ugagaucaugCgaaaacugcuuu...............   1     1    Fm1
......................................................................ugagaucaugGgaaaacugcuuu...............   2     1    Fm1
......................................................................ugagaucaugugaaaacugcuuuA..............   14    1    Fm1
......................................................................ugagaucaugugaaaacAgcuuuu..............   1     1    Fm1
......................................................................ugagaucauUugaaaacugcuuuu..............   1     1    Fm1
......................................................................ugagaucaugugaaaaGugcuuuu..............   1     1    Fm1
......................................................................ugagaucaugugaaaacGgcuuuu..............   2     1    Fm1
......................................................................ugagaucaugugaaaacugcuuCu..............   4     1    Fm1
......................................................................ugagaucaugugaaaacugcuuAu..............   21    1    Fm1
......................................................................ugagaucaugugaaaaAugcuuuu..............   2     1    Fm1
......................................................................ugagaucaugugaaaacugcuuuu..............   392   0    Fm1
......................................................................ugagaucaugugaaaacCgcuuuu..............   2     1    Fm1
......................................................................ugagauAaugugaaaacugcuuuu..............   1     1    Fm1
......................................................................ugagaucaugugaaaacugcuuuuU.............   21    1    Fm1
.......................................................................gagaucaugugaaaacugcu.................   3     0    Fm1
.......................................................................gagaucaugugaaaacugcuu................   7     0    Fm1
.......................................................................gagaucaugugaaaacugcuuu...............   3     0    Fm1
.......................................................................gagaucaugugaaaacugcuuuu..............   2     0    Fm1
.......................................................................gagaucaugugaaaacugcuuuA..............   1     1    Fm1
.........................................................................gaucaugugaaaacugcuu................   3     0    Fm1
```
